# Genome-wide next-generation DNA and RNA sequencing reveals a mutation that perturbs splicing of the phosphatidylinositol glycan anchor biosynthesis class H gene (*PIGH*) and causes arthrogryposis in Belgian Blue cattle

**DOI:** 10.1186/s12864-015-1528-y

**Published:** 2015-04-18

**Authors:** Arnaud Sartelet, Wanbo Li, Eric Pailhoux, Christophe Richard, Nico Tamma, Latifa Karim, Corinne Fasquelle, Tom Druet, Wouter Coppieters, Michel Georges, Carole Charlier

**Affiliations:** GIGA-R & Department of Animal Sciences, Unit of Animal Genomics, Faculty of Veterinary Medicine, University of Liège, Avenue de l’Hôpital 1, 4000 Liège, Belgium; INRA, UMR 1198, Biologie du Développement et Reproduction, F-78350 Jouy-en-Josas, France; GIGA Genomic Platform, GIGA, University of Liège, Avenue de l’Hôpital 1, 4000 Liège, Belgium

**Keywords:** Arthrogryposis syndrome, *PIGH* gene, Splice-site mutation, Glycosylphosphatidyl inositol deficiency, Belgian Blue Cattle breed

## Abstract

**Background:**

Cattle populations are characterized by regular outburst of genetic defects as a result of the extensive use of elite sires. The causative genes and mutations can nowadays be rapidly identified by means of genome-wide association studies combined with next generation DNA sequencing, provided that the causative mutations are conventional loss-of-function variants. We show in this work how the combined use of next generation DNA and RNA sequencing allows for the rapid identification of otherwise difficult to identify splice-site variants.

**Results:**

We report the use of haplotype-based association mapping to identify a locus on bovine chromosome 10 that underlies autosomal recessive arthrogryposis in Belgian Blue Cattle. We identify 31 candidate mutations by resequencing the genome of four cases and 15 controls at ~10-fold depth. By analyzing RNA-Seq data from a carrier fetus, we observe skipping of the second exon of the *PIGH* gene, which we confirm by RT-PCR to be fully penetrant in tissues from affected calves. We identify - amongst the 31 candidate variants - a C-to-G transversion in the first intron of the *PIGH* gene *(c211-10C > G)* that is predicted to affect its acceptor splice-site. The resulting PIGH protein is likely to be non-functional as it lacks essential domains, and hence to cause arthrogryposis.

**Conclusions:**

This work illustrates how the growing arsenal of genome exploration tools continues to accelerate the identification of an even broader range of disease causing mutations, therefore improving the management and control of genetic defects in livestock.

**Electronic supplementary material:**

The online version of this article (doi:10.1186/s12864-015-1528-y) contains supplementary material, which is available to authorized users.

## Background

The extensive use of elite sires exacerbated by the large-scale exploitation of artificial insemination in cattle breeding causes important reductions in effective population size and the common spread of loss-of-function variants. This in turn is responsible for the periodic outburst of genetic defects that cause considerable economic loss and welfare issues. With the development of genome-wide SNP arrays for all livestock species, it has become possible to rapidly map the underlying locus by means of autozygosity mapping to intervals that typically span 2 to 5 megabases thereby proving the inherited nature and mode of inheritance of the corresponding condition (f.i. [1]). With the advent of targeted or whole-genome next generation sequencing (NGS), it is becoming increasingly facile to identify the causative mutation, needed to develop accurate diagnostic tests, provided that the mutation is a frame-shift, nonsense, canonical splice-site, or severe missense variant. In other cases, the causative mutation may remain elusive for a considerably longer time. We show in this work how the combined use of DNA and RNA NGS data, may accelerate the discovery of an otherwise elusive, novel class of causative mutations.

## Results and discussion

### Arthrogryposis emerges as a new genetic defect in Belgian Blue Cattle

**Figure 1:**
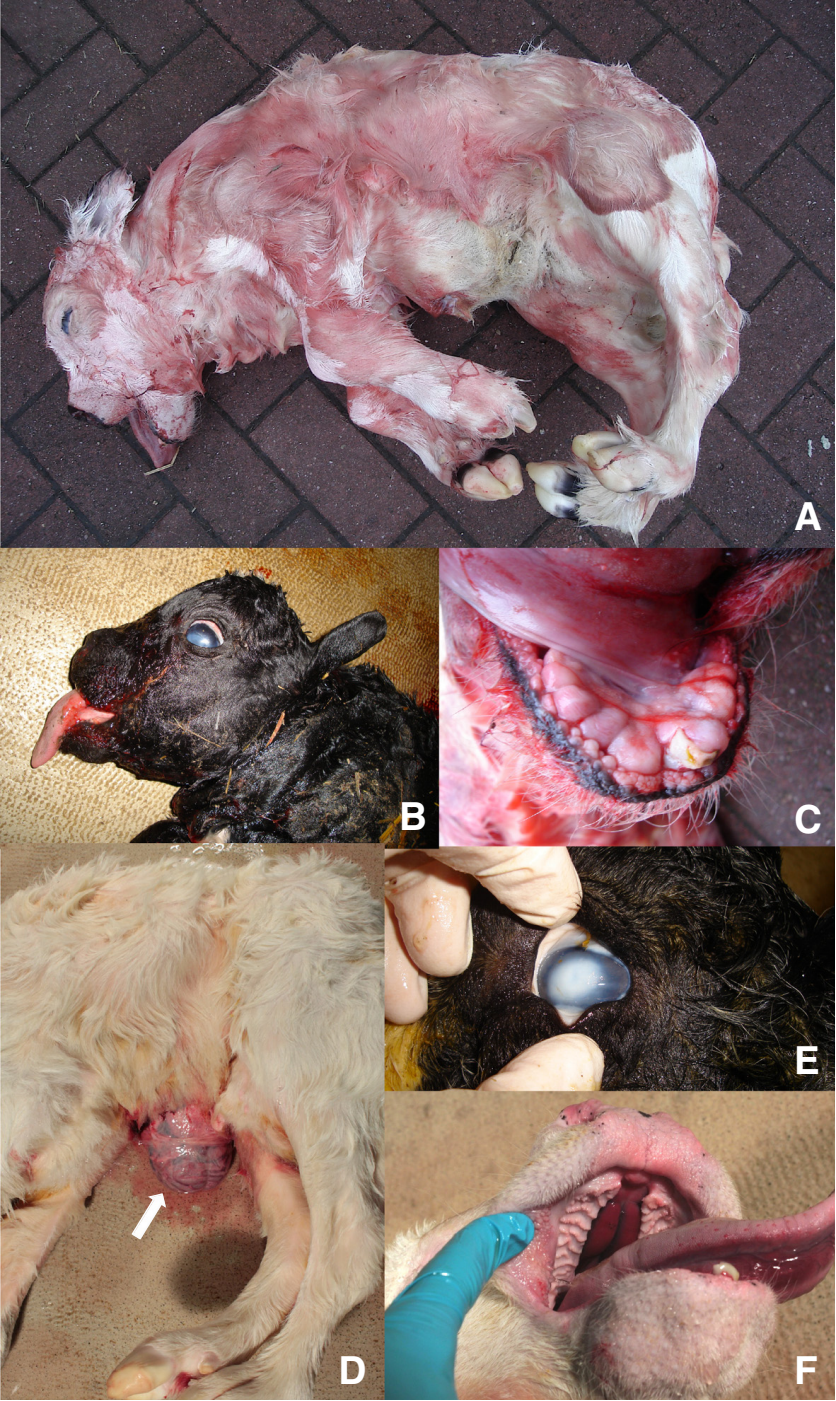
Lethal arthrogryposis syndrome clinical spectrum. **A**. Generalized arthrogryposis. **B**. Brachygnathism and macroglossy. **C**. Impaired tooth eruption. **D**. Omphalocoele. **E**. Corneal clouding. **F**. Hard cleft palate.

We recently established an “heredo-surveillance platform” to effectively identify and control inherited defects that recurrently emerge as a result of intensive use of elite sires in Belgian Blue and other cattle breeds (f.i. [1]). Twenty-five Belgian-Blue cases of a new form of arthrogryposis were referred to this platform in 2009 alone. Affected calves were all characterized by arthrogryposis (hooked joints) of the four limbs, severe scoliosis (curved spine), and a stocky head with macroglossy and impaired tooth eruption. A majority of cases suffered from cleft palate (20/25) and upper lip (3/25), omphalocele (abdominal wall defect with umbilical hernia; 19/25) and corneal clouding (21/25) (Figure 1). Several dams developed metritis and peritonitis, caused by hydrops (accumulation of excessive fluid in the allantoïc or amniotic space) of the fetal membranes due to impaired fetal swallowing.

### A haplotype-based GWAS maps the culprit locus to a 2.2 Mb interval on bovine chromosome 10

**Figure 2:**
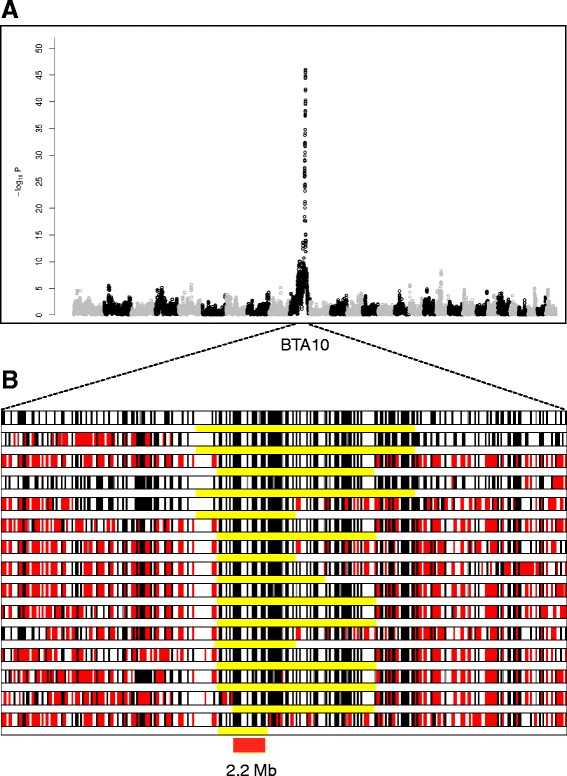
Genetic mapping of the mutation causing the arthrogryposis syndrome in Belgian Blue Cattle. **A**. Manhattan plot for the case–control GWAS study. **B**. Genotypes of 15 cases for a BTA10 segment centered around the most significant GWAS peak, and encompassing 324 SNP (from 70 to 90 Mb). Homozygous genotypes are shown in black or white, heterozygous genotypes in red. The presumed ancestral haplotype encompassing the mutation is underlined in yellow. The 2.2 Mb region of homozygosity shared by all cases is highlighted in red.

The 25 cases traced back, on sire and dam side, to the artificial insemination (AI) sire *Kalimine du Barsy Fontaine*, suggesting autosomal recessive inheritance. We therefore genotyped 15 cases with the bovine SNP50 beadchip (Illumina, San Diego) to perform a genome-wide association study (GWAS). We used the genotypes from 275 Belgian Blue AI sires, obtained with the bovine HD (700 K) beadchip (Illumina, San Diego), as controls. The analysis was restricted to 34,368 SNPs shared by both arrays, and conducted with GLASCOW as previously described [2]. This yielded a single genome-wide significant signal (p = 10^−40^) on chromosome 10 (Figure 2A). It resulted from autozygosity of the 15 cases for a 2.2 Mb (chr10:78,424,435-80,602,211 bp; *Bos taurus* assembly: BosTau6/UMD3) identical-by-descent haplotype, hence confirming the suspected mode of inheritance (Figure 2B).

### Resequencing the whole genome of four cases identifies 31 candidate causative mutations

The corresponding interval is collinear with a 2.4 Mb segment of human chromosome 14 (chr14: 66,474,214-68,918,385 bp) and encompasses 22 annotated genes. As none of these would be obvious candidates, we generated paired-end libraries for four cases and re-sequenced them to average 2.5-fold depth (for a combined total coverage of ~10 fold) on a Illumina GAIIX instrument as described [3]. Equivalent genomic sequences of 15 healthy Belgian Blues that did not carry the incriminated haplotype were used as controls. Sequence reads were mapped on the BosTau6/UMD3 reference genome using BWA [4], and variants called with SAMTools [5]. We detected 2,968 variants with quality score > 100 mapping to the 2.2 Mb interval. As expected, the four cases appeared homozygous for the 21 Beadchip SNPs defining the associated haplotype. Filtering against known bovine dbSNP SNPs and variants observed in at least one of the 15 controls, and demanding homozygosity for the four cases, left us with only 31 candidate variants (Additional file 1). Yet, none of these would alter coding sequences (missense or nonsense), or map within three base pairs from an exon-intron junction.

### RNASeq reveals skipping of PIGH exon 2 and pinpoints an intronic mutation as the likely causative variant

**Figure 3:**
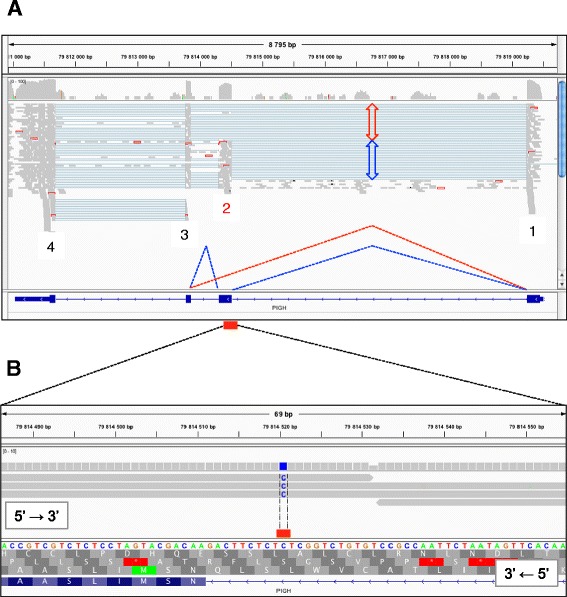
Alternative splicing at the *PIGH* locus and private *c211-10C > G* intronic variation. **A**. Screen capture of an IGV output from liver RNASeq data of a heterozygote mutant embryo aligned on the bovine genomic reference sequence at the *PIGH* locus (top). The four exons appear as stacks of grey reads and splicing is schematically denoted by thin blue lines. Complete skipping of *PIGH* exon 2 in ~ half of the transcripts is noticeable (red versus blue arrow height). *PIGH* intron/exon annotation and the two alternative splicing events are represented by dashed red (skipped exon 2) and blue (incorporated) lines. **B**. Screen capture of an IGV output displaying (i) on the positive strand (top, 5′ to 3′), a private G to C mutation (blue) from genomic DNA sequence reads of four pooled homozygous cases, (ii) on the negative strand (bottom, 3′ to 5′), *PIGH* intron 1/exon 2 annotation showing the private mutation position at −10 nucleotide in the splice acceptor sequence (*c211-10C > G*, red bar).

The findings described in the previous paragraph suggested that the causative mutation was either regulatory or affecting splicing otherwise. To pursue this hypothesis, we took advantage of available RNASeq data (cfr. M&M) from liver (83 Mb uniquely aligned) and cerebral cortex (105 Mb) of a 60-day post fertilization Belgian Blue fetus shown by SNP genotyping to carry the arthrogryposis risk haplotype. Sequence reads were analyzed using TopHat and Cufflinks [6] and predicted transcripts mapping to the arthrogryposis locus visualized in the IGV browser [7]. We readily noticed skipping of the second exon of approximately half (20/45) of the *PIGH* (phosphatidylinositol glycan anchor biosynthesis class H) transcripts (Figure 3A). To verify whether this observation might be related to the arthrogryposis condition we extracted RNA from available skeletal muscle and kidney of an affected calf and an age-matched control, and performed RT-PCR using primers located in exon 1 and 4 of the *PIGH* gene. While we obtained a unique band of expected 593-bp size for the control, the only band obtained from the case RNA was 377~bp (Additional file 2). Sequencing of the corresponding RT-PCR products revealed the expected *PIGH* exon 1-2-3 sequence for the controls, yet two distinct sequences for the case. The most abundant (~75%) form corresponded to the skipping of exon 2, while the minor (~25%) form was - in addition to missing exon 2 – devoid of the first AAG triplet of exon 3 (Additional file 2). Note the 3′ AG end of this lysine codon susceptible to act as a cryptic acceptor splice-site. This form was also observed in five RNASeq reads, validating this finding (data not shown).

Interestingly, one of the 31 candidate mutations identified by whole genome sequencing is a C-to-G transversion located in the first intron of *PIGH*, 10-bp upstream of the exon 2 junction *″211-10C > G*) (Figure 3B). The corresponding residue is located in the consensus polypyrimidine track defining canonical “GT-AG” type acceptor splice-sites (f.i. [8]), and is conserved in 26 of the 27 sequenced mammals (Additional file 3). We developed a 5′exonuclease assay to interrogate the *c211-10C > G* variant and genotyped 25 cases, 21 parents and > 10,000 healthy Belgian Blue animals. All cases were homozygous “GG”, and all parents “CG” as expected. Six percent of the healthy Belgian Blues were carriers, while none were homozygous “GG” (p = 0.00263).

### The clinical spectrum of arthrogryposis is compatible with severe glycosylphosphatidyl inositol (GPI) deficiency

The PIGH protein is ubiquitously expressed. It is one of seven highly conserved (from yeast to human) subunits of the complex catalyzing the first step out of eleven in the biosynthesis of the glycophosphatidyl inositol (GPI) anchor, a complex C-terminal posttranslational modification concerning > 150 proteins or ~0.5% of cellular proteins (http://www.uniprot.org/uniprot) (f.i. [9-11]). Within the GPI-GlcNAc transferase complex (GPI-GnT), PIGH is anchored in the membrane of the endoplasmic reticulum (ER) by two trans-membrane helices (residues 38–55 and 60–78), connected by a short ER intra-luminal loop (56–59), and bounded by a short N-terminal (1–37) and a long C-terminal cytoplasmic tail (79–188) [12]. Deleting exon 2 generates a protein missing amino-acids 61–130, therefore unlikely to be properly anchored in the ER membrane and to be functional (Additional file 4). In culture, cells with mutations in *PIGH* do not display any GPI-anchored protein (GPI-AP) surface expression (f.i. [13]). In animals, all known GPI-anchor related defects characterized by a complete lack of GPI-anchored proteins are lethal for the embryo. It is thought that this embryonic-lethal phenotype is due to the fact that several cell-to-cell adhesion molecules required for normal development and acting at specific developmental stages are GPI-anchored (reviewed in [11]). The additional file 5 lists the known GPI-AP and their associated knock-out phenotype in mice. Taken together, our results strongly suggest that the observed *PIGH c211-10C > G* mutation causes arthrogryposis in Belgian Blue Cattle.

## Conclusions

We herein describe the identification of an intronic mutation that disrupts *PIGH* function by causing the skipping of exon 2, thereby causing a severe and lethal form of arthrogryposis in homozygous animals. We mapped the corresponding locus by performing a haplotype-based GWAS, an approach that has proven successful in many instances (f.i. [1]). If the causative mutation is either a stop-gain, frameshift, splice-site variant within 2-bp from an exon-intron boundary, or a severe missense variant, its identification is often relatively straightforward. In our experience, this situation occurs approximately fifty percent of the time, which is in agreement with the data reported by [14]. In the other cases, the identification of the causative mutation may be considerably more arduous. In the present study, it was the concomitant generation of RNASeq data from tissue of a carrier animal that provided the clues for the identification of the causative mutation. The detection of exon skipping in an essential gene that was not observed in non-carrier animals pointed towards one of 31 genetically defined mutations (located in the immediate vicinity of the skipped exon) as being the likely causative mutation. It would have been very difficult to predict the effect of this variant, located at 10-bp from the exon-intron junction, on the splicing reaction without the information on the corresponding transcripts. Our work therefore illustrates the value of the combined availability of DNA and RNASeq data to rapidly identify a broader panel of disease causing mutations.

Although we consider the supporting evidence to be very strong, we haven’t formally proven that skipping of exon 2 of the *PIGH* gene abrogates its function and interferes with GPI-anchor biosynthesis. In human and mice, functional validation of GPI biosynthesis pathway’s defects relies on the detection of incomplete or absent cell surface expression of CD59, a well-characterized GPI-anchored protein (f.i. [12]). This quantification is typically performed by FACS on fresh erythrocytes. Unfortunately, homozygous mutant calves are dead at birth, precluding the collection of fresh blood. Producing and collecting homozygous mutant fetuses by mating carriers was beyond the scope of this study.

To the best of our knowledge, this the first report of a naturally occurring loss-of-function mutation in the *PIGH* gene in animals. A genetic test interrogating the *PIGH c211-10C > G* variant has been developed and offered to breeders since 2011. Its widespread use has led to the rapid elimination of this syndrome from the Belgian Blue population.

## Methods

### Ethics statement

Blood samples were collected from sires, cows and calves, by trained veterinarians following standard procedures and relevant Belgian national guidelines. For bovine fetuses production and collection, experiments reported in this work are in agreement with the ethical guidelines of the French National Institute for Agricultural Research (INRA). Fetuses were produced by artificial insemination of a wild-type Belgian Blue female with semen of a Belgian Blue confirmed-carrier male, 7-days old embryos recovered, transferred to recipient females in an INRA experimental farm (France), then collected at 60 days post-fertilization at the INRA slaughterhouse (France). The protocol (N°: 12/046) was approved by the local ethical committee (COMETHEA) and Eric Pailhoux is the recipient of an official authorization for animal experimentation (N°: B91-649).

### SNP array Genotyping

Genomic DNA of cases was extracted from 350 μl of blood using the MagAttract DNA Blood Midi M48 Kit (Qiagen). Genomic DNA of controls was extracted from frozen semen using the MagAttract Mini M48 Kit (Qiagen). The 15 cases of the initial genome scan were genotyped using a custom-made 50 K SNP array [1]. The 275 control sires were genotyped with the BovineHD BeadChip (Illumina). SNP genotyping was conducted using standard procedures at the GIGA genomics core facility.

### RNAseq data generation and analysis

As part of a companion project, bovine embryos were produced by directed mating between a sire carrier for the arthrogryposis haplotype and a wild-type cow. A panel of tissues - including pituitary, cerebral cortex and liver - from two embryos diagnosed as carrier based on a haplotype test, were collected at 60 days post fertilization (dpf); total RNA was extracted and cDNA librairies were produced using the TrueSeq mRNA kit from Illumina, following manufacturer’s instructions. Equal amounts of each indexed library were combined and sequenced 2X100bp on one lane of a HighSeq2000 instrument. Transcriptomes were analyzed using the RNASeq tool kit TopHat and Cufflinks [6]. Mapped RNASeq reads for the carrier embryo were visually evaluated in IGV (Integrative Genome Browser) [7].

### Mutation validation at the mRNA level in case and control calves

Total RNA was extracted from kidney and muscle of one homozygous case and one unaffected unrelated individual using Trizol (Invitrogen) following manufacturer’s instructions. The obtained total RNA was treated with TurboDNaseI (Ambion) and cDNA was synthesized using Superscript™III First Strand Synthesis System for RT-PCR (Invitrogen). A 593 bp *PIGH* cDNA fragment was PCR amplified using a specific primers respectively located in exon 1 (PIGH_UP : 5′-TCT CTT TGC GCT CGC TCA CC-3′) and exon 4 (PIGH_DN: 5′-GAT CCA CCA CAT CCA TAC TGG-3′). Amplicons were directly sequenced using the Big Dye terminator cycle sequencing kit (Applied Biosystems, Foster City, CA). Electrophoresis of purified sequencing reactions was performed on an ABI PRISM 3730 DNA analyzer (PE Applied Biosystems, Foster City, CA). Sequence traces were aligned and compared to bovine cDNA sequence using the Phred/Phrap/Consed package (http://www.phrap.org/phredphrapconsed.html).

### 5′ exonuclease diagnostic assay of the PIGH c211-10C > G splice acceptor site mutation

A 5′ exonuclease assay was developed to genotype the *PIGH c211-10C > G* mutation, using 5′- ATG GCA GCA GAG AGG ATC ATG -3′ and 5′- GGA GTT GAC TTA TTA ACC AGC AGA GA -3′ as PCR primers, and 5′-TGT CTG GCT **[C]**TC TCT TC-3′ (wild type *C* allele) and 5′-TCT GGC T**[G]**T CTC TTC -3′ (mutant *G* allele) as probes (Taqman, Applied Biosystems, Fosters City, CA). Reactions were carried out on an ABI7900HT instrument (Applied Biosystems, Fosters City, CA) using standard procedures.

### Availability of supporting data

All the variations of the IBD disease haplotype have been submitted to dbSNP (http://www.ncbi.nlm.nih.gov/SNP/). They will be publicly available with the next dbSNP Build (B145), planned for fall, 2015. Additional files 6 and 7 provide respectively the list of “ss” numbers, genomic positions (*Bos taurus* assembly: BosTau6/UMD3) and the list of corresponding sequence variations.

## Additional files

Additional file 1:
**Chromosomal position and sequence context for the disease risk haplotype private mutations within the IBD interval.** Are given, from left to right: the chromosome (Chr.), the variant start position on UMD3.1/bosTau6 assembly (Start), the reference sequence allele (Ref.), the derived allele (Der.), both on the positive strand, the underlying gene (Gene) if any, the regional annotation (Annotation) and the variant sequence context, within a unique sequence (Unique, yes) or in a repetitive region (Unique, no). Additional file 2:
**Effect of the** ***c211-10C > G*** **mutation at the RNA level.** A. Partial schematic representation of the *PIGH* organization (genomic and mRNA) accompanied by wild-type cDNA sequence traces across exon 1/exon 2 and exon 2/exon 3 junctions obtained respectively with a forward primer in exon 1 and a reverse primer in exon 4. B. Corresponding agarose gel showing cDNA amplification products from kidney (K) for a wild-type (WT) animal, from kidney (K) and skeletal muscle (SM) for a mutant (MUT) and for a RT-minus control (RT-); MW: molecular weight marker (SmartLadder, Eurogentec). C. Mutant cDNA sequence traces obtained from the ~ 377 bp amplification product with reverse (top) and forward (bottom) primers respectively; the cryptic “AAG” acceptor site in exon 3 is highlighted in dark orange; height of depicted white and orange bars represents the ratio between the two mutant splice forms, with total skipping of exon 2 in white and additional skipping of a “AAG” triplet in orange. Additional file 3:
***PIGH*** **first exon acceptor splice-site evolutionary conservation.** Alignment and conservation of genomic DNA sequences corresponding to the *PIGH* gene (final part of intron 1 in lower cases and beginning of coding exon 2 in upper cases) is presented for placental mammals, genomic sequences were downloaded from the UCSC genome browser; bovine wild-type (WT) “C” nucleotide (nt) is highlighted in black and corresponding mutant (MUT: *c211-10C > G*) “G” nt in orange; consensus acceptor splice-site sequence, from intronic nt −12 to nt −1, is depicted below the alignment (with Y for C/T; N for A/T/C/G and essential acceptor splice-site (AG) at positions −2 and −1 in black); for every consensus position, nucleotide usage (A/U(T)/C/G in %) is presented; one can notice that at nt position −10, “G” nt is present in only 6% (orange) of the acceptor splice-site sequences (SpliceDB: Burset et al., [8]). Additional file 4:
**PIGH protein evolutionary conservation and topology in the endoplasmic reticulum (ER) plasma membrane.** A. Protein alignment of the PIGH protein from mammals to yeast; the two transmembrane (TM) domains, as annotated in UniProtKB, are underlined and highlighted in yellow; wild-type (WT) and mutant (MUT) bovine PIGH are presented and the deduced missing amino acid sequence - corresponding to the second TM domain and part of the cytoplasmic C-terminal domain - is boxed in grey. B. Schematic representation of the WT PIGH protein (left), adapted from Watanabe et al. [12] confirmed by the topological prediction obtained with TMHMM2.0 software (screen capture of the TMHMM result, right) [15]; putative topology for the MUT protein is shown accordingly (bottom); TM domains are colored in yellow. Additional file 5:
**GPI-anchored proteins reviewed in mice and specific associated knock-out phenotypes (if any).** Are given from left to right: accession number in UniProtKB (Entry); gene symbol (Entry name); reviewed or unreviewed status (Status); full protein name (Protein name); alternative gene symbols (Gene names); amino acid number (Length); knock-out if any (Knock-out, KO); phenotype severity in homozygous KO (Phenotype severity); KO phenotype as described in MGI (Mouse Genome Informatics; http://www.informatics.jax.org/marker/). Additional file 6
**List of genomic positions and corresponding “ss” (Submitted SNP) numbers for sequence variations of the IBD disease haplotype.**
Additional file 7:
**List of genomic positions, flanking sequences and corresponding sequence variations of the IBD disease haplotype.**
