# Supplementary material for: Genome-wide next-generation DNA and RNA sequencing reveals a mutation that perturbs splicing of the phosphatidylinositol glycan anchor biosynthesis class H gene (PIGH) and causes arthrogryposis in Belgian Blue cattle
Source: BMC Genomics. 2015 Apr 18;16(1):316. doi: 10.1186/s12864-015-1528-y (PMC4404575; doi:10.1186/s12864-015-1528-y)
Supplement: Additional file 1: — Chromosomal position and sequence context for the disease risk haplotype private mutations within the IBD interval. Are given, from left to right: the chromosome (Chr.), the variant start position on UMD3.1/bosTau6 assembly (Start), the reference sequence allele (Ref.), the derived allele (Der.), both on the positive strand, the underlying gene (Gene) if any, the regional annotation (Annotation) and the variant sequence context, within a unique sequence (Unique, yes) or in a repetitive region (Unique, no). [file 12864_2015_1528_MOESM1_ESM.pdf]

Additional file 1: Chromosomal position and sequence context for the disease risk haplotype private mutations within the IBD interval.

Chr.	Start	Ref.	Der.	Gene	Annotation	Unique
chr10	78427250	G	A	/	intergenic	no
chr10	78454747	A	C	/	intergenic	no
chr10	78674184	T	G	/	intergenic	no
chr10	78714805	C	G	/	intergenic	yes
chr10	78951178	C	T	*GPHN*	intronic	no
chr10	78974848	G	A	*GPHN*	intronic	no
chr10	79151521	T	A	*GPHN*	intronic	yes
chr10	79151943	C	G	*GPHN*	intronic	yes
chr10	79160268	GT	GTT	*GPHN*	intronic	no
chr10	79162630	C	T	*GPHN*	intronic	yes
chr10	79168827	G	C	*GPHN*	intronic	yes
chr10	79170470	C	T	*GPHN*	intronic	yes
chr10	79171445	CTT	C	*GPHN*	intronic	yes
chr10	79172280	A	C	*GPHN*	intronic	no
chr10	79176708	T	A	*GPHN*	intronic	yes
chr10	79181403	GTTACTTA	GTTA	*GPHN*	intronic	yes
chr10	79203039	G	C	*GPHN*	intronic	no
chr10	79234207	G	T	*GPHN*	intronic	no
chr10	79275933	A	G	*GPHN*	intronic	no
chr10	79453112	T	C	*FAM71D*	intronic	yes
chr10	79695337	C	T	*TMEM229B*	intronic	no
**chr10**	**79814520**	**G**	**C**	***PIGH***	**intronic**	**yes**
chr10	80188354	T	G	*ZFYVE26*	intronic	no
chr10	80194440	T	C	*ZFYVE26*	intronic	no
chr10	80225126	G	A	*ZFYVE26*	intronic	yes
chr10	80231100	C	T	*ZFYVE26*	intronic	yes
chr10	80248464	A	C	/	intergenic	no
chr10	80354103	A	G	*RAD51B*	intronic	no
chr10	80486651	C	T	*RAD51B*	intronic	yes
chr10	80502656	C	T	*RAD51B*	intronic	yes
chr10	80515466	G	A	*RAD51B*	intronic	yes
