# Supplementary figures and images for: Genome-wide next-generation DNA and RNA sequencing reveals a mutation that perturbs splicing of the phosphatidylinositol glycan anchor biosynthesis class H gene (PIGH) and causes arthrogryposis in Belgian Blue cattle
Source: BMC Genomics. 2015 Apr 18;16(1):316. doi: 10.1186/s12864-015-1528-y (PMC4404575; doi:10.1186/s12864-015-1528-y)

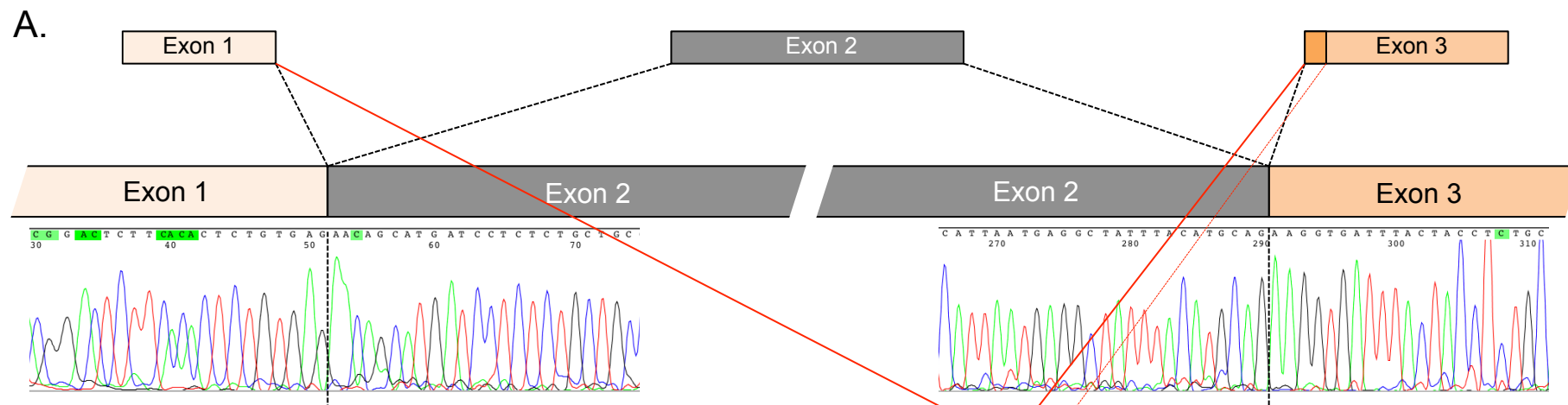

**B.**

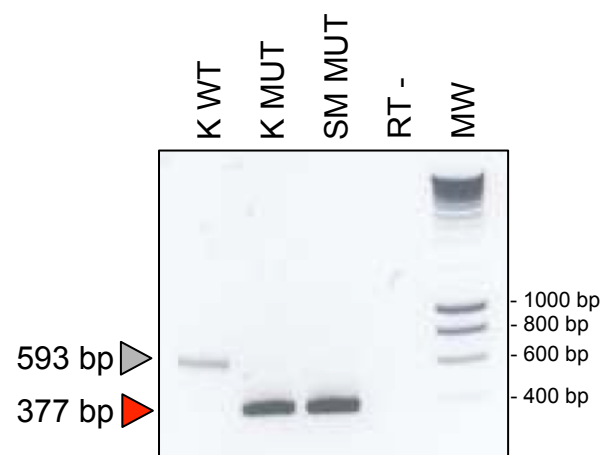

**C.**

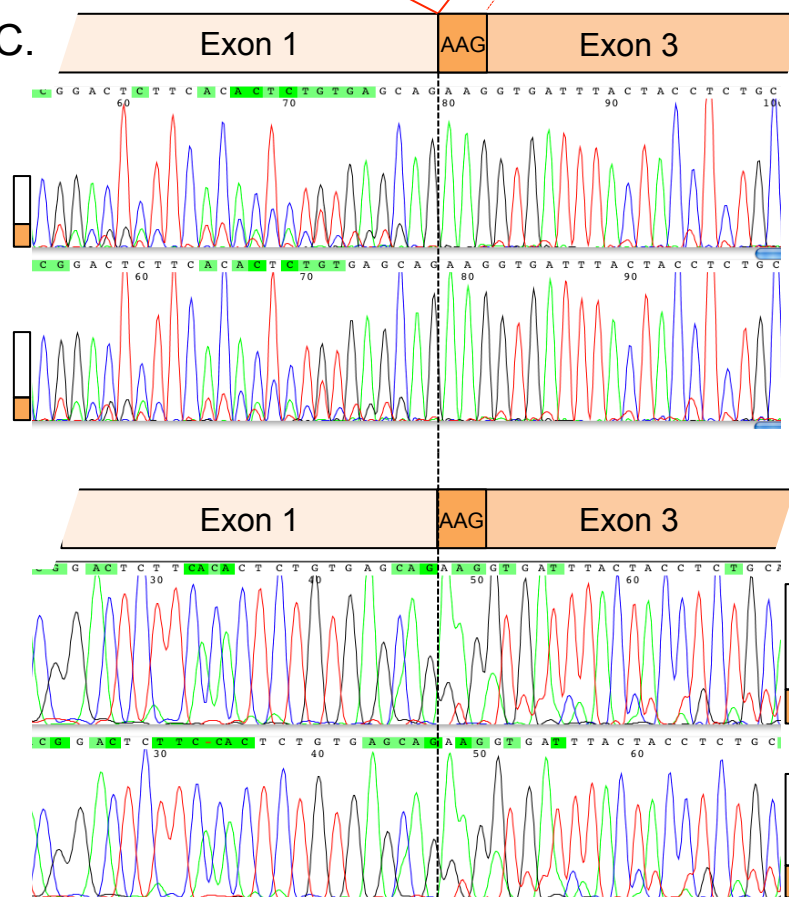

Supplement: Additional file 2: — Effect of the c211-10C > G mutation at the RNA level. A. Partial schematic representation of the PIGH organization (genomic and mRNA) accompanied by wild-type cDNA sequence traces across exon 1/exon 2 and exon 2/exon 3 junctions obtained respectively with a forward primer in exon 1 and a reverse primer in exon 4. B. Corresponding agarose gel showing cDNA amplification products from kidney (K) for a wild-type (WT) animal, from kidney (K) and skeletal muscle (SM) for a mutant (MUT) and for a RT-minus control (RT-); MW: molecular weight marker (SmartLadder, Eurogentec). C. Mutant cDNA sequence traces obtained from the ~ 377 bp amplification product with reverse (top) and forward (bottom) primers respectively; the cryptic “AAG” acceptor site in exon 3 is highlighted in dark orange; height of depicted white and orange bars represents the ratio between the two mutant splice forms, with total skipping of exon 2 in white and additional skipping of a “AAG” triplet in orange. [file 12864_2015_1528_MOESM2_ESM.pdf]

A.

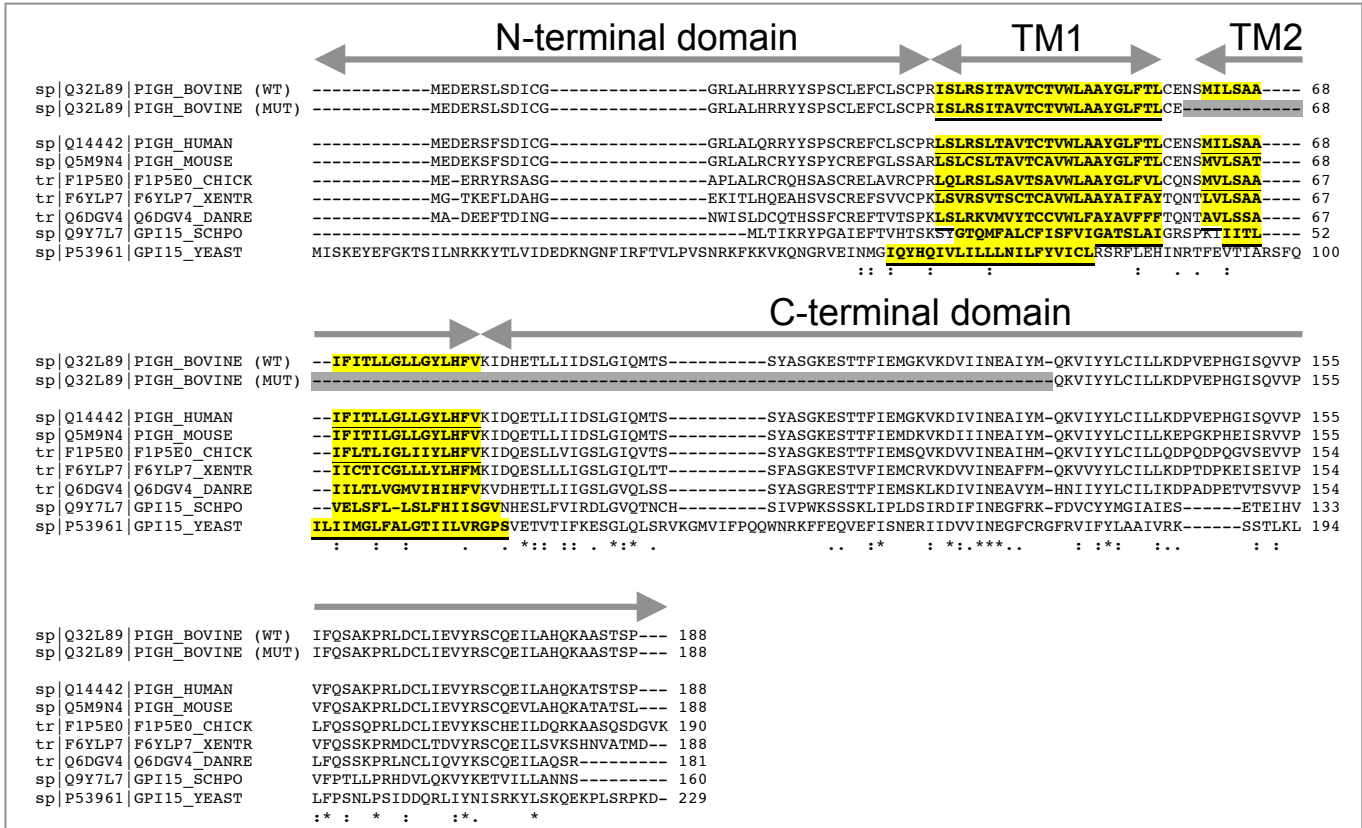

B.

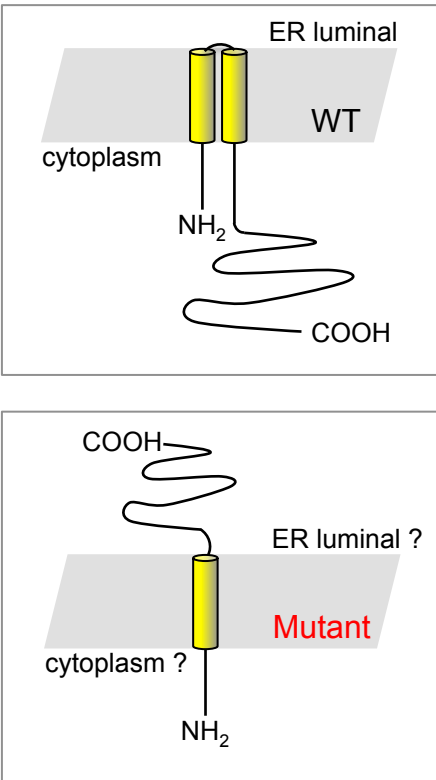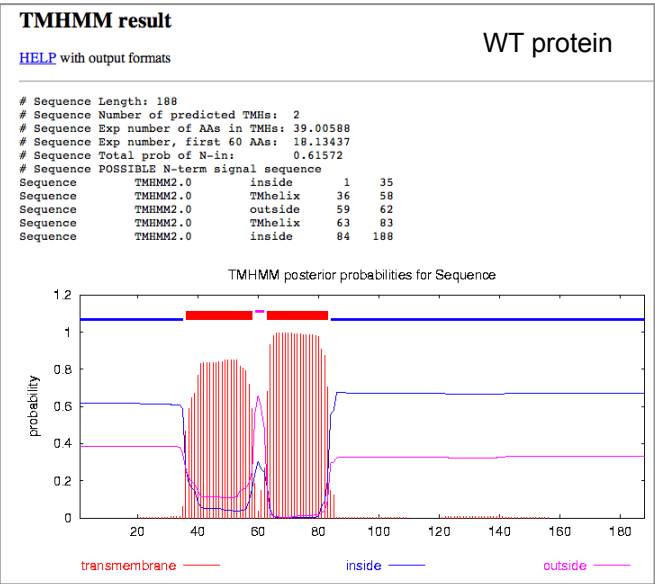

Supplement: Additional file 4: — PIGH protein evolutionary conservation and topology in the endoplasmic reticulum (ER) plasma membrane. A. Protein alignment of the PIGH protein from mammals to yeast; the two transmembrane (TM) domains, as annotated in UniProtKB, are underlined and highlighted in yellow; wild-type (WT) and mutant (MUT) bovine PIGH are presented and the deduced missing amino acid sequence - corresponding to the second TM domain and part of the cytoplasmic C-terminal domain - is boxed in grey. B. Schematic representation of the WT PIGH protein (left), adapted from Watanabe et al. [12] confirmed by the topological prediction obtained with TMHMM2.0 software (screen capture of the TMHMM result, right) [15]; putative topology for the MUT protein is shown accordingly (bottom); TM domains are colored in yellow. [file 12864_2015_1528_MOESM4_ESM.pdf]
