# Supplementary material for: Genome-wide next-generation DNA and RNA sequencing reveals a mutation that perturbs splicing of the phosphatidylinositol glycan anchor biosynthesis class H gene (PIGH) and causes arthrogryposis in Belgian Blue cattle
Source: BMC Genomics. 2015 Apr 18;16(1):316. doi: 10.1186/s12864-015-1528-y (PMC4404575; doi:10.1186/s12864-015-1528-y)
Supplement: Additional file 3: — PIGH first exon acceptor splice-site evolutionary conservation. Alignment and conservation of genomic DNA sequences corresponding to the PIGH gene (final part of intron 1 in lower cases and beginning of coding exon 2 in upper cases) is presented for placental mammals, genomic sequences were downloaded from the UCSC genome browser; bovine wild-type (WT) “C” nucleotide (nt) is highlighted in black and corresponding mutant (MUT: c211-10C > G) “G” nt in orange; consensus acceptor splice-site sequence, from intronic nt −12 to nt −1, is depicted below the alignment (with Y for C/T; N for A/T/C/G and essential acceptor splice-site (AG) at positions −2 and −1 in black); for every consensus position, nucleotide usage (A/U(T)/C/G in %) is presented; one can notice that at nt position −10, “G” nt is present in only 6% (orange) of the acceptor splice-site sequences (SpliceDB: Burset et al., [8]). [file 12864_2015_1528_MOESM3_ESM.pdf]

Additional file 3

	INTRON 1																	EXON 2								
hg18	t	c	t	g	g	c	t	c	t	c	t	c	t	t	c	a	g	A	A	C	A	G	C	A	T	G
panTro2	t	c	t	g	g	c	t	c	t	c	t	c	t	t	c	a	g	A	A	C	A	G	C	A	T	G
gorGor1	t	c	t	g	g	c	t	c	t	c	t	c	t	t	c	a	g	A	A	C	A	G	C	A	T	G
ponAbe2	t	c	t	g	g	c	t	c	t	c	t	c	t	t	c	a	g	A	A	C	A	G	C	A	T	G
rheMac2	t	c	t	g	g	c	t	c	t	c	t	c	t	t	c	a	g	A	A	C	A	G	C	A	T	G
calJac1	t	c	t	g	g	c	t	c	t	c	t	c	t	t	c	a	g	A	A	C	A	G	C	A	T	G
tarSyr1	t	c	t	g	g	c	t	c	t	-	-	c	t	t	c	a	g	A	A	C	A	G	C	A	T	G
tupBel1	t	c	t	g	g	c	t	c	t	c	t	c	t	t	c	a	g	A	A	C	A	G	C	A	T	G
dipOrd1	t	c	t	g	g	-	-	c	t	c	t	t	t	t	c	a	g	A	A	C	A	G	C	A	T	G
mm9	t	c	t	g	g	c	t	c	t	c	t	c	t	t	c	a	g	A	A	T	A	G	C	A	T	G
rn4	t	c	t	g	g	c	t	c	t	c	t	c	t	t	c	a	g	A	A	T	A	G	C	A	T	G
speTri1	t	c	t	g	g	c	t	c	t	c	t	c	t	t	c	a	g	A	A	C	A	G	C	A	T	G
oryCun1	t	c	t	g	g	c	t	c	t	g	t	c	t	t	c	a	g	A	A	C	A	G	C	A	T	G
vicPac1	t	c	t	g	g	c	t	c	t	c	t	c	t	t	c	a	g	A	A	C	A	G	C	A	T	G
bosTau4 WT	t	c	t	g	g	c	t	**c**	t	c	t	c	t	t	c	a	g	A	A	C	A	G	C	A	T	G
bosTau4 MUT	t	c	t	g	g	c	t	**g**	t	c	t	c	t	t	c	a	g	A	A	C	A	G	C	A	T	G
turTru1	t	c	t	g	g	c	t	c	t	-	-	c	t	t	c	a	g	A	A	C	A	G	C	A	T	G
equCab2	t	c	t	g	g	c	t	c	t	c	t	c	t	t	t	a	g	A	A	C	A	G	C	A	T	G
pteVam1	t	c	t	g	g	c	t	c	t	c	t	c	t	t	c	a	g	A	A	C	A	G	C	A	T	G
myoLuc1	t	c	t	g	g	c	t	c	t	c	t	c	t	t	c	a	g	A	A	C	A	G	C	A	T	G
sorAra1	t	c	t	g	c	t	-	c	t	c	c	t	t	c	c	a	g	A	A	C	A	G	C	A	T	G
eriEur1	t	t	t	g	g	c	t	c	t	-	-	t	t	t	c	a	g	A	A	C	A	G	C	A	T	G
felCat3	t	c	t	g	g	c	t	c	t	c	t	c	t	t	c	a	g	A	A	C	A	G	C	A	T	A
canFam2	t	c	t	g	g	c	t	c	t	c	t	c	t	t	c	a	g	A	A	C	A	G	C	A	T	G
cavPor3	t	c	t	g	g	c	t	c	t	t	t	c	t	t	c	a	g	A	A	C	A	G	C	A	C	G
ochPri2	t	c	c	g	g	c	t	t	t	g	c	c	t	t	c	a	g	A	A	C	A	G	C	A	T	G
micMur1	t	c	t	g	g	c	t	c	t	c	t	c	t	t	c	a	g	A	A	C	A	G	C	A	T	G
otoGar1	t	c	t	g	g	c	t	c	t	c	t	c	t	t	c	a	g	A	A	C	A	G	C	A	T	G
proCap1	t	c	t	g	g	c	t	c	t	c	t	c	t	t	c	a	g	A	A	C	A	G	C	A	T	G
	*			*					*				*			*	*	*	*		*	*	*	*		

consensus splice site	Y	Y	**Y**	Y	Y	Y	Y	Y	N	Y	**A**	**G**
	-12	-11	-10	-9	-8	-7	-6	-5	-4	-3	-2	-1
% of A	15	6	15	11	19	12	3	10	25	4	**100**	0
% of U	53	60	49	49	45	45	57	58	29	31	0	0
% of C	21	24	**30**	33	28	36	36	28	22	65	0	0
% of G	10	10	6	7	9	7	7	5	24	1	0	**100**
